# An efficient molecular genetic testing strategy for incontinentia pigmenti based on single-tube long fragment read sequencing

**DOI:** 10.1038/s41525-024-00421-z

**Published:** 2024-05-29

**Authors:** Min Chen, Mei-Hua Tan, Jiao Liu, Yan-Mei Yang, Jia-Ling Yu, Li-Juan He, Ying-Zhi Huang, Yi-Xi Sun, Ye-Qing Qian, Kai Yan, Min-Yue Dong

**Affiliations:** 1 Women’s Hospital, School of Medicine, Zhejiang University, Hangzhou, Zhejiang 310006 P. R. China; 2 Key Laboratory of Reproductive Genetics (Zhejiang University), Ministry of Education, Hangzhou, Zhejiang 310006 P. R. China; 3 Key Laboratory of Women’s Reproductive Health of Zhejiang Province, Hangzhou, Zhejiang 310006 P. R. China; 4 BGI Genomics, Shenzhen, Guangdong, 518083 P. R. China; 5 Lishui Maternity and Child Health Care Hospital, Lishui, Zhejiang 323000 P. R. China

**Keywords:** Genetic testing, Genetics research

## Abstract

Incontinentia pigmenti (IP) is a rare X-linked dominant neuroectodermal dysplasia that primarily affects females. The only known causative gene is *IKBKG*, and the most common genetic cause is the recurrent *IKBKG*^△4–10^ deletion resulting from recombination between two MER67B repeats. Detection of variants in *IKBKG* is challenging due to the presence of a highly homologous non-pathogenic pseudogene *IKBKGP1*. In this study, we successfully identified four pathogenic variants in four IP patients using a strategy based on single-tube long fragment read (stLFR) sequencing with a specialized analysis pipeline. Three frameshift variants (c.519-3_519dupCAGG, c.1167dupC, and c.700dupT) were identified and subsequently validated by Sanger sequencing. Notably, c.519-3_519dupCAGG was found in both *IKBKG* and *IKBKGP1*, whereas the other two variants were only detected in the functional gene. The *IKBKG*^△4–10^ deletion was identified and confirmed in one patient. These results demonstrate that the proposed strategy can identify potential pathogenic variants and distinguish whether they are derived from *IKBKG* or its pseudogene. Thus, this strategy can be an efficient genetic testing method for *IKBKG*. By providing a comprehensive understanding of the whole genome, it may also enable the exploration of other genes potentially associated with IP. Furthermore, the strategy may also provide insights into other diseases with detection challenges due to pseudogenes.

## Introduction

Incontinentia pigmenti (IP, OMIM 308300), also known as Bloch-Sulzberger syndrome, is a rare neuroectodermal dysplasia characterized by various abnormalities of the skin, hair, teeth, eyes, and central nervous system^1^. It occurs primarily in females and can cause in utero lethality in males. Skin abnormalities in affected females evolve through four stages from infancy to adulthood: bullous stage, verrucous stage, hyperpigmentation stage, and atretic stage^2^. The hyperpigmented lesions typically fade after puberty. Other clinical features include alopecia, hypodontia, retinal hypervascularization, seizures, and central nervous system anomalies^3,4^. Occasionally, some affected males survive, which may be due to the presence of an additional X chromosome (47,XXY), somatic mosaicism, or hypomorphic variants^5^.

IP is caused by variants in the *IKBKG* (also known as *NEMO*) gene on Xq28. The gene is ~23 kb in length and consists of 10 exons. The detection rate of pathogenic variants is ~80%^6,7^. Deletions of exons 4–10, *IKBKG*^△4–10^, account for the majority of identified variants^8,9^. The complexity of variant detection is due to a highly homologous non-pathogenic pseudogene, *IKBKGP1*, which is located 31 kb distal to *IKBKG* in the opposite orientation^10^. In contrast to *IKBKG*, deletion of exons 4–10 in *IKBKGP1* does not cause symptoms^11^. In addition, the existence of two 879 bp repeats, termed MER67B, one in intron 3 (MER67B^1st^) and one downstream of exon 10 (MER67B^2nd^), further complicates the diagnosis. Despite the advances in sequencing technology, molecular testing for IP is still mainly based on long-range polymerase chain reaction (PCR) and multiplex ligation-dependent probe amplification (MLPA)^3,12^. Therefore, alternative efficient approaches are needed to identify *IKBKG*-specific variations. Single-tube long fragment read (stLFR, MGI Tech) has been reported as an efficient technology that can utilize next-generation sequencing (NGS) platforms to sequence long DNA molecules^13^. By co-barcoding, short reads from the same long DNA fragment can be assembled according to the barcodes. The stLFR can retain long-range genomic information of ~20–300 kb in length. It may thus provide a solution for addressing complex genetic issues, such as identifying large structural variations (SVs) and distinguishing functional genes from pseudogenes. However, its related clinical applications have not yet been fully demonstrated.

Here, we present an efficient strategy for genetic testing of IP based on stLFR sequencing. We applied this strategy to four IP families and successfully identified four pathogenic variants in the *IKBKG* gene. The new strategy enables us to detect putative pathogenic variants throughout the genome and can differentiate specific variants of *IKBKG* from *IKBKGP1*. Thus, the strategy proposed here is able to overcome pseudogene-related complications in IP detection.

## Results

### stLFR read properties and analysis results using routine stLFR pipelines

**Fig. 1:**
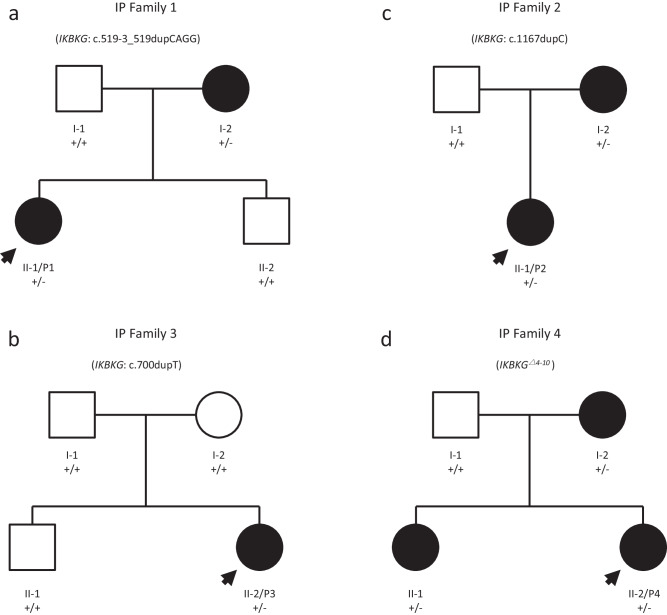
Pedigrees of four IP families with the identified variants. Affected individuals are shown in black, while arrows indicate the probands in each family (**a**–**d**). The genotype of each evaluated individual is displayed below his or her symbol. A *plus sign* denotes the normal allele, and a *minus sign* denotes the mutant allele.

**Fig. 2:**
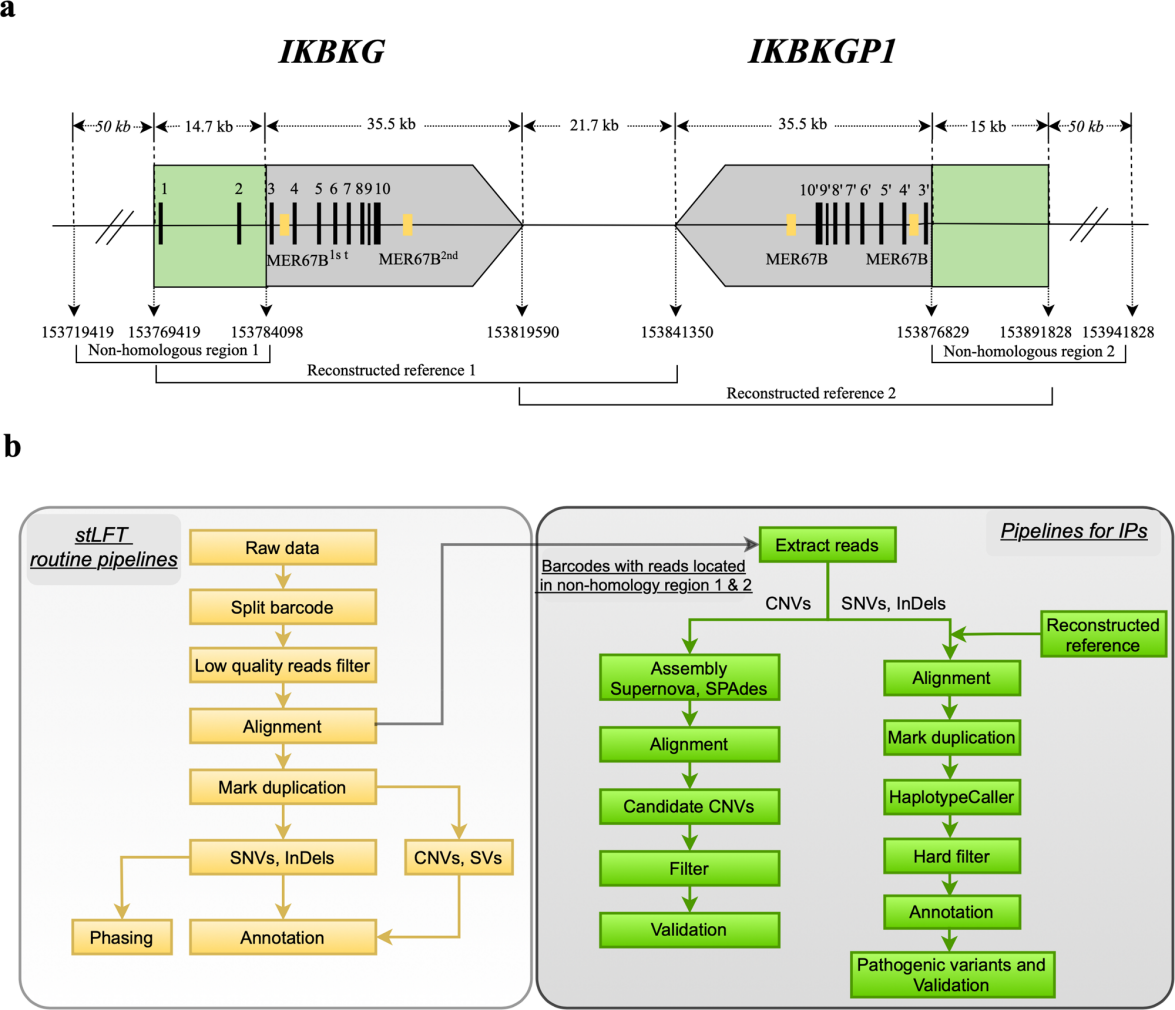
Genomic characteristics of *IKBKG* and its detection analysis strategy. **a** Schematic representation of the *IKBKG* gene and its downstream pseudogene *IKBKGP1*. The *IKBKG* gene contains 10 exons (exons 1–10), while *IKBKGP1* contains only 7 exons (exons 3–10). The gray square arrows represent the highly homologous regions, and the green boxes represent the non-homologous regions. The MER67B repeated sequences are shown as yellow boxes. **b** Diagram of the analysis pipelines. The left side shows the routine bioinformatic pipeline of stLFR, and the right side shows a specialized analysis pipeline for IP.

Four patients (Fig. 1) were detected using a strategy based on stLFR sequencing with a specialized analysis pipeline (Fig. 2). The quality of stLFR sequencing data was summarized in Table 1. The average genome sequencing depth was ~20×, ranging from 19.98× to 22.47×. The average long fragment length of the four samples was 39.29 kb, 22.41 kb, 35.4 kb, and 48.48 kb, respectively. Based on the routine analysis of stLFR, >3 million SNPs, >800 thousand insertion-deletion mutations (indels), and hundreds of copy number variants (CNVs) were identified for each sample, and none of them was located in the genomic region related to IP.

**Table 1:** Statistical results of stLFR sequencing quality and variants detection

Sample ID	P1	P2	P3	P4
Library statistics				
Total bases sequenced (Gb)	109.9	107.02	105.05	114.27
Average genome sequencing depth	20.3	20.89	19.98	22.47
Duplicate rate (%)	30.72	24.9	21.25	34.43
Coverage at least 4X (%)	98.22	98.23	98.15	98.43
Barcode number	49,119,117	56,301,677	60,170,592	12,950,168
Average fragments per barcode	1.47	1.70	1.59	1.47
Average co-barcoded reads per fragment	31.79	21.85	24.13	37.89
Fragment number	19,154,096	29,292,350	25,101,016	19,036,172
Average fragment length (bp)	39287.7	22413.1	35401.7	48481.3
Average fragment read number	21.57	12.89	15.13	25.78
SNP calls				
Total SNPs	3,833,760	3,769,397	3,734,632	3,689,342
Fraction of SNPs in dbSNP (%)	97.98	98.19	98.18	98.36
Fraction of SNPs in 1000 Genomes (%)	90.74	92.56	92.66	95.33
Novel SNPs (Fraction%)	76,807	67,579	67,201	60,544
Indel calls				
Total indels	852137	841609	822643	847858
Fraction of indels in dbSNP (%)	85.04	85.91	86.44	87.04
Fraction of indels in 1000 Genomes (%)	54.32	54.93	55.56	53.17
Novel indels (Fraction%)	123,650	117,846	110,829	109,764
CNV calls				
Total CNVs	241	243	246	885
Duplication CNVs	61	66	63	293
Deletion CNVs	180	177	183	592

### Single nucleotide variations (SNVs) and indels detection by a specialized analysis pipeline

**Fig. 3:**
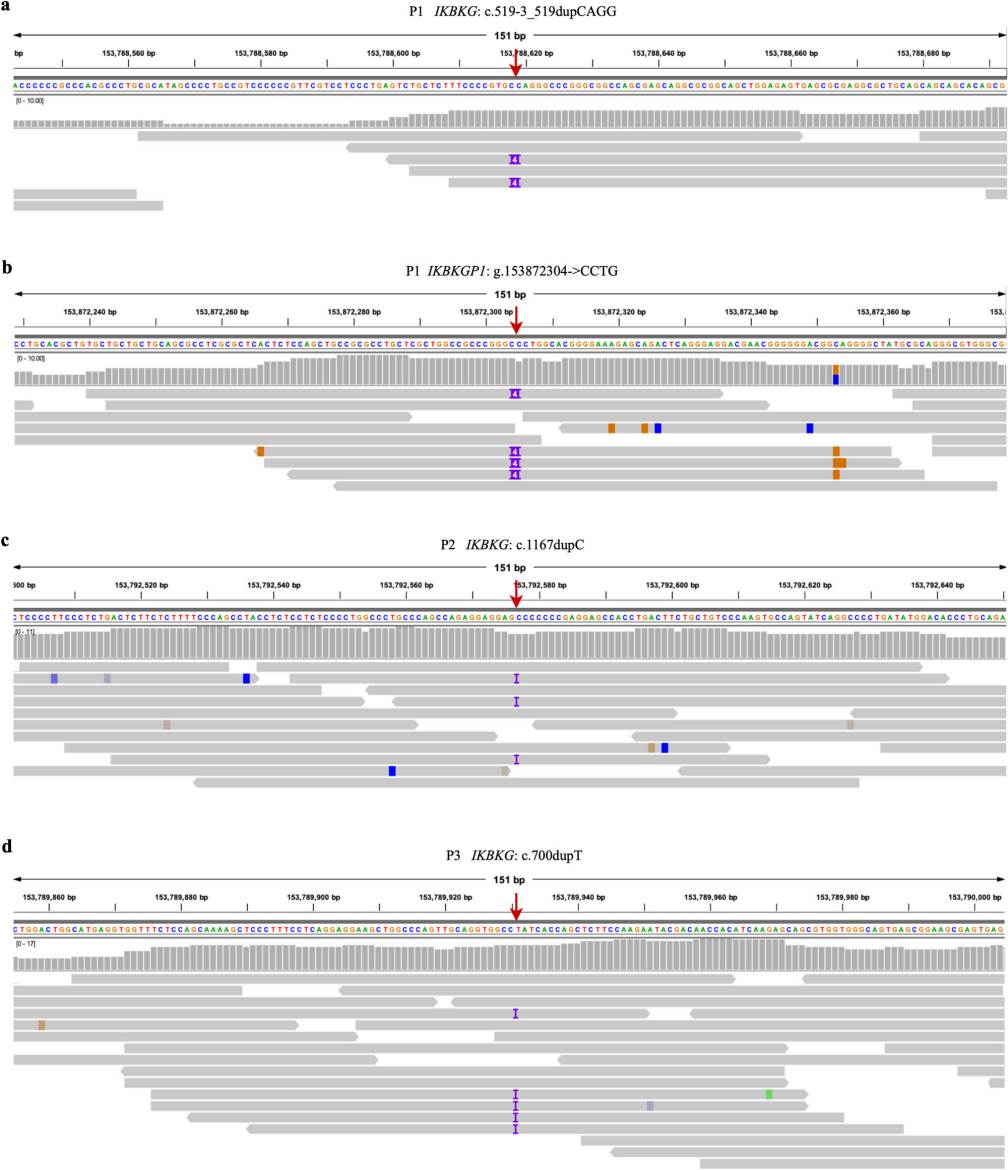
IVG visualized diagram of three disease-associated variants in three patients. The c.519-3_519dupCAGG variant is found in both *IKBKG* (**a**) and *IKBKGP1* (**b**) in P1. The c.1167dupC and c.700dupT variants are present only in the *IKBKG* gene in P2 (**c**) and P3 (**d**), respectively.

**Fig. 4:**
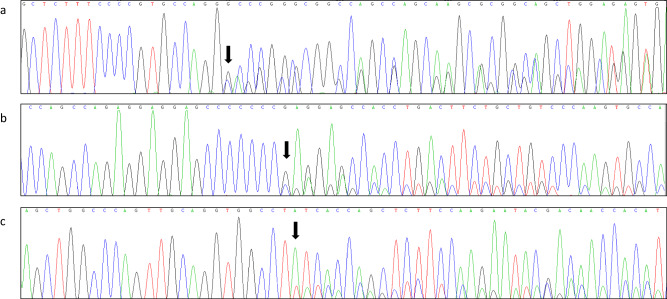
Sanger sequencing validation results of three frameshift variants. Variants are indicated by arrows. **a** c.519-3_519dupCAGG in P1; **b** c.1167dupC in P2; **c** c.700dupT in P3.

After filtering with a specialized analysis pipeline, three intragenic variants were found in the *IKBKG* gene (NM_001099857.5: c.519-3_519dupCAGG, c.1167dupC and c.700dupT) (Fig. 3 and Table 2). Considering the inheritance pattern, allele frequency, predicted impact, and the annotation results of VarSome, these three variants were considered potential pathogenic variants for patients P1-P3. Notably, variant c.519-3_519dupCAGG was found in both *IKBKG* and *IKBKGP1* in P1, whereas the other two variants were only detected in the functional gene. Subsequent variant testing was performed in the probands and their family members by Sanger sequencing (Figs. 1 and 4). In IP families 1 and 2, both the proband and the mother carried the variant, suggesting maternal transmission. In family 3, the variant proved to be de novo.

**Table 2:** Potential pathogenic variants identified in the *IKBKG* gene (NM_001099857.5)

Sample ID	Chromosome	Start	Stop	Call	Gene symbol	c.HGVS	p.HGVS	Zygosity	Pathogenicity (VarSome)
P1	chrX	153788618	153788618	CAGG	IKBKG	c.519-3_519dupCAGG	p.(A174Qfs*15)	Het	Pathogenic
P2	chrX	153792576	153792576	C	IKBKG	c.1167dupC	p.(E390Rfs*5)	Het	Pathogenic
P3	chrX	153789930	153789930	T	IKBKG	c.700dupT	p.(Y234Lfs*20)	Het	Likely pathogenic

These frameshift variants were predicted to cause the loss of normal protein function either through protein truncation or nonsense-mediated mRNA decay. Two of them (c.519-3_519dupCAGG and c.1167dupC) have been previously reported^7,14,15^, and published functional studies have shown that c.1167dupC would cause a damaging effect on protein function^16,17^. None of these variants were observed in the gnomAD database. Therefore, they were considered pathogenic or likely pathogenic.

### CNV detection by supernova and SPAdes

**Fig. 5:**
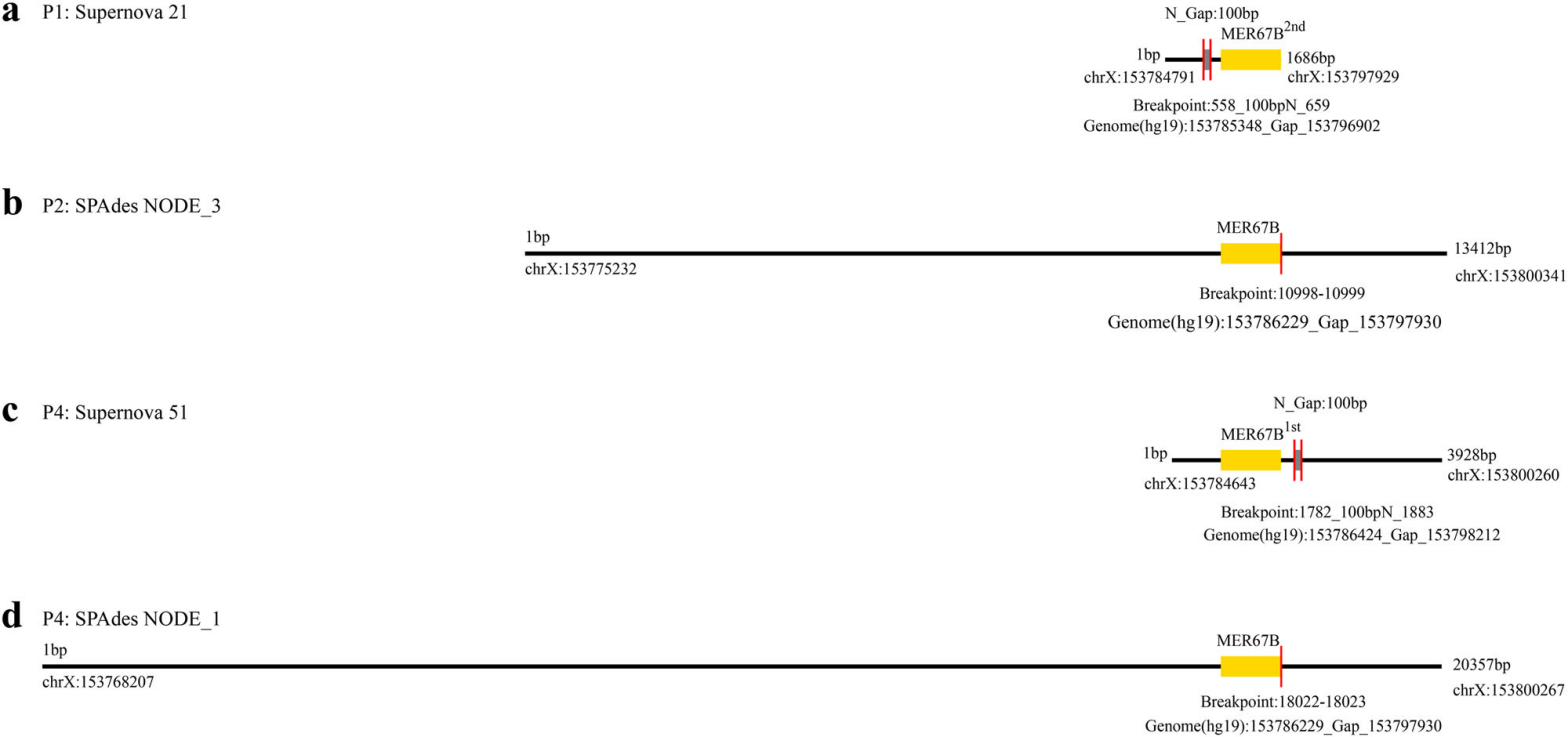
The breakpoints and recombination of MER67B in the assembly results of candidate CNVs. Scaffolds detected by Supernova or SPAdes in P1 (**a**), P2 (**b**) and P4 (**c** and **d**). The horizontal lines represent the assembly sequences, and the red vertical lines represent the breakpoints. The gray rectangles are the N-gap regions, and the yellow rectangles are the recombined MER67B region. The genomic position (hg19) of the breakpoint is shown below each contig.

All assembly results of Supernova and SPAdes were demonstrated in Supplementary Figure 1. Two scaffolds (scaffold 21 in P1 and scaffold 51 in P4) with breakpoints near MER67B in *IKBKG* were detected by Supernova. They were subsequently excluded because the breakpoints were caused by N-gaps (Fig. 5). With SPAdes, two discontinuous or misassembled assemblers between two MER67B repeats were detected (NODE_3 in P2 and NODE_1 in P4) (Supplementary Figure 1 and Fig. 5). Compared to Supernova, the assemblers of SPAdes were longer, and no N was present. To further determine the authenticity of these two assemblers, reads passing through the breakpoint were extracted for long DNA fragment analysis. In P2, one of six reads was aligned to the MER67B1st sequence (Ref MER67B1st), and the remaining five were aligned to either the *IKBKG*^△4–10^ mutant sequence (Mut) or the MER67B2nd sequence (Ref MER67B2nd) (Supplementary Figure 2a). Because *IKBKG*^△4–10^ is caused by recombination between two MER67B repeat sequences, the reads supporting assemblers at the breakpoint could be a true deletion signal or simply mapped to Ref MER67B2nd. In P4, five extracted reads were mapped to Ref MER67B1st, and the other five were mapped to either Mut or Ref MER67B2nd (Supplementary Figure 3a). To verify the validity of these reads, we further used the co-barcoding information to trace all reads of these long DNA fragments. The distribution of all co-barcoded reads was illustrated in Supplementary Fig. 2b and 3b. In P2, multiple reads were mapped to the deletion region in three long fragments, demonstrating that there was indeed no deletion in these fragments. The remaining two fragments (1439_459_1531 and 851_274_1146) were considered as potential *IKBKG*^△4–10^, due to the absence of reads in the deletion region. In P4, three fragments turned out to be aligned to Ref MER67B^2nd^ due to the presence of co-barcoded reads in the deletion region, while the other two fragments (531_1256_485 and 221_57_1365) were considered as potential *IKBKG*^△4–10^ (Supplementary Figure 3b). No CNVs were found in *IKBKGP1* (data not shown).

**Fig. 6:**
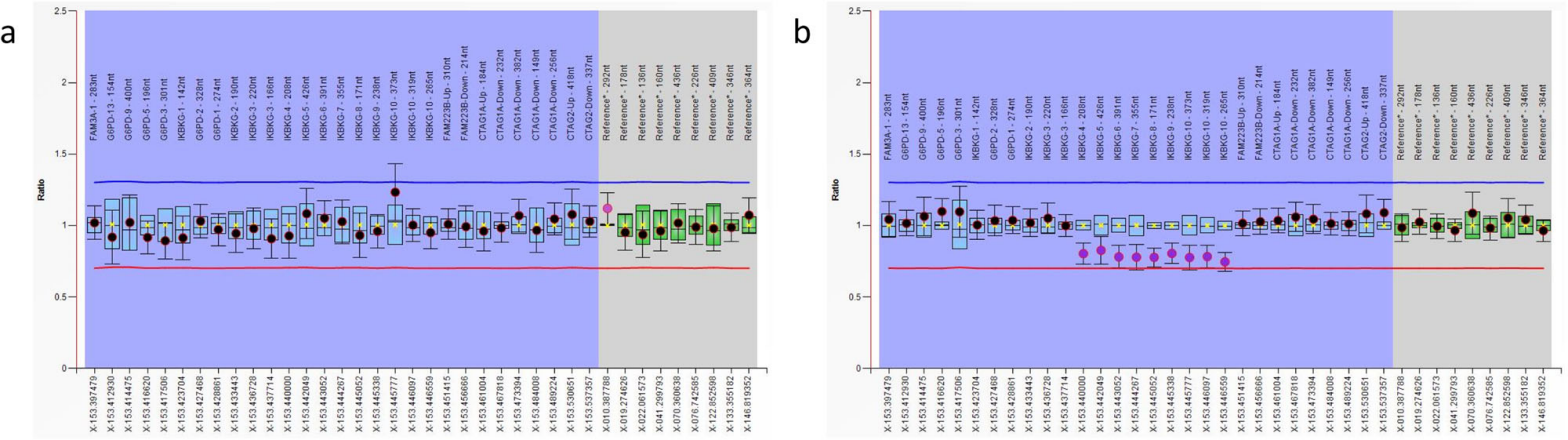
MLPA validation of candidate pathogenic CNVs. **a**, **b** are MLPA results of P2 and P4, respectively.

The *IKBKG*^△4–10^ deletions identified in P2 and P4 were then validated by MLPA (Fig. 6). The deletion in P2 turned out to be a false positive result, while the deletion in P4 was confirmed to be true. Since MLPA could not determine whether the deletion was in *IKBKG* or *IKBKGP1*, long-range PCR followed by Sanger sequencing was performed. The results showed that the deletion was in the *IKBKG* gene (data not shown). Combined with the results of stLFR analysis, *IKBKG*^△4–10^ in P4 was an *IKBKG*-specific deletion. MLPA confirmed that P4’s mother and sister also carried *IKBKG*^△4–10^ (data not shown).

## Discussion

Currently, the main challenge in IP detection is to differentiate the true *IKBKG* gene from its pseudogene *IKBKGP1*. To identify SNVs in the *IKBKG* gene, a common approach is to test all exons, exon-surrounding intronic regions, and promoter regions separately^3,10^. To eliminate the *IKBKGP1* gene, a first round of long-range PCR would be performed^18–20^ (Supplementary Table 1). An alternative approach to identify variants in the functional *IKBKG* gene is direct sequencing of the cDNA^16,19,21^. However, sequencing each fragment can be a tedious task, and variants in regulatory, deep intronic, or non-coding regions remain undetected. Although NGS is widely used in clinical molecular testing, its application in IP is limited. Due to a 35.5 kb homologous region shared by *IKBKG* and *IKBKGP1*^10^, it is extremely difficult for short-read technologies to identify specific variants. Attempts have been made using long-range PCR followed by NGS^6,22^. For CNVs, nested long-range PCR remains the gold standard method^10,23,24^. Another commonly used method is MLPA, which, however, cannot differentiate between *IKBKG* and its pseudogene^3^. Therefore, there is still a need to develop efficient testing approaches that can identify variants in both exons and introns. To fill the gaps, we proposed a strategy using stLFR in combination with unique barcodes to retrieve long fragment information. Routine analysis pipelines of stLFR can provide a comprehensive picture of the whole genome. Besides SNVs and CNVs, indels and other SVs can also be detected. However, routine analysis cannot identify variants in the homologous region of *IKBKG* due to mapping quality values of 0. Therefore, we established a specialized analysis pipeline for *IKBKG*. All putative pathogenic variants in the functional *IKBKG* gene can be detected and distinguished from *IKBKGP1*. Our data further showed that three of these variants (c.1167dupC, c.700dupT, and *IKBKG*^△4–10^) were present only in the *IKBKG* gene, and one (c.519-3_519dupCAGG) was present in both *IKBKG* and *IKBKGP1*. Additionally, our work may facilitate the discovery of novel putative variants or genes. Approximately one-fifth of all cases still have no known cause^7,25^. Individuals may have low-level somatic mosaicism^6^, pathogenic variants in other regions of the *IKBKG* gene^7^, or even in other genes. Our strategy can detect variants in these regions more effectively than conventional methods. Given that *IKBKG* is the only gene associated with IP and that some patients have a milder or different clinical phenotype^6,26^, investigation of other potential genetic causes should be considered. Although little data was used to analyze the *IKBKG* and *IKBKGP1* genes, the remaining data could be used to identify other SVs and other causative genes. One drawback of stLFR is the cost, but it could provide a more comprehensive understanding of genome structural features and overcome the interference of highly homologous pseudogenes in the molecular testing of IPs. This, in turn, may improve IP detection and is worth considering.

In our proposed strategy, two assembly software were used to detect CNVs. Supernova is one of the most commonly used software for de novo assembly of linked reads and is capable of generating diploid assemblies of the human genome^27,28^. However, our results showed that Supernova would easily introduce Ns into the assembly results, making it difficult to detect the target CNVs. This may be due to the limited amount of data for assembly contigs. The sequencing depth of genomes in previous studies is generally more than 60X^29–31^, while it was about 20X in our study. Increasing the amount of sequencing data may improve the ability of Supernova to detect *IKBKG*^△4–10^. SPAdes, on the other hand, is a flexible assembler that is suitable for various data formats generated by different sequencing platforms and is capable of generating long and accurate assembly results from cross-species raw sequencing reads^32,33^. Although the amount of data was limited, our study showed that SPAdes successfully identified *IKBKG*^△4–10^. According to the MLPA verification results, the deletion in P4 was confirmed, while the one in P2 was a false positive. Although it may be frustrating to require MLPA and long-range PCR for verification, the strategy itself is logical. Insufficient data and low-coverage reads of long DNA fragments are possible causes. With the continuous reduction of sequencing costs, the specificity and positive predictive value of the pipeline for detecting assembly CNVs could be improved by increasing the amount of sequencing data.

In conclusion, we have proposed a feasible and promising strategy for IP testing using stLFR with a specialized analysis pipeline. It could provide a comprehensive understanding of the whole genome, and all putative pathogenic variants of IP could be detected. More importantly, specific variants of *IKBKG* could be distinguished from *IKBKGP1*. In addition, our strategy has the potential to uncover additional genes that may be associated with IP. The performance of our pipeline for IP testing could be further improved with the decreasing cost of sequencing. Furthermore, the strategy proposed here can address pseudogene-related issues in IP testing and provide insights into other diseases with detection challenges due to pseudogenes.

## Methods

### Patients and their clinical manifestations

Five patients from four families were clinically diagnosed with IP based on typical skin manifestations (Fig. 1). P1 was further confirmed by skin biopsy. Both mothers of P1 and P2 had very mild hyperpigmentation. P3 had no family history. The mother of P4 had mild blisters at birth. Unilateral amblyopia and agenesis of permanent teeth occurred in one patient (P1), while one patient developed blindness in the right eye (P4). None of them exhibited any neurological disorders.

Written informed consent was obtained to participate and publish from both parents and patients. This study was conducted in accordance with the Declaration of Helsinki and was approved by the Ethics Committee at Women’s Hospital, School of Medicine, Zhejiang University (20190038). The study is compliant with the Guidance of the Ministry of Science and Technology (MOST) for the Review and Approval of Human Genetic Resources.

### stLFR library preparation and sequencing

The genomic DNA was extracted from peripheral blood leukocytes using the QIAGEN MagAttract HMW DNA Kit (QIAGEN, Germany) following the manufacturer’s protocol. An stLFR library was constructed using the MGIEasy stLFR Library Prep Kit (MGI Tech, China) according to the manufacturer’s instructions. Briefly, transposons were inserted into long DNA molecules. Subsequently, these transposon-inserted DNA sequences were hybridized with clonal barcoded beads and then ligated with barcoded oligo and adapters via splint oligo. After adding the library adapters, PCR amplification and circularization were performed to generate DNA nanoballs (DNBs). The prepared library was then sequenced on the MGISEQ-2000 platform (MGI Tech) with a 100 bp paired-end strategy. The expected raw data of each sample is 100GB or more.

### Primary data analysis by routine stLFR pipelines

The routine bioinformatic pipelines of stLFR sequencing were summarized in Fig. 2. Briefly, raw sequencing reads were first demultiplexed with the associated barcodes of long DNA molecules using the barcode split tool (GitHub). Routine pre-processing of the raw reads was then conducted, including filtering of low-quality reads, alignment (software: BWA, reference genome: GRCh37/HG19), and elimination of PCR duplicates. Variants, including SNVs, indels, CNVs, and SVs, were detected and annotated based on WGS pipelines using the MegaBOLT system (MGI Tech).

### SNVs and indels detection of targeted genes based on reconstructed references

New reference genomes were constructed according to the genomic characteristics (Fig. 2a). Non-homologous regions and an additional 50 kb of extended regions were used to extract co-barcoded sequencing reads of long DNA fragments, which were designated as “non-homologous region 1” and “non-homologous region 2” for *IKBKG* and *IKBKGP1*, respectively. Sequence related to *IKBKG* was designated as reconstructed reference 1, while sequence related to *IKBKGP1* was designated as reconstructed reference 2. Based on the primary data analysis, a specialized analysis pipeline was developed to distinguish the functional *IKBKG* gene from its pseudogene *IKBKGP1* (Fig. 2b). Variants were detected by GATK HaplotypeCaller and filtered with hard filter patterns (Supplementary methods). The reconstructed reference positions were then converted to hg19 for subsequent annotation analysis. Finally, variants were annotated using unpublished in-house software. Annotated information included basic variant information, protein functional hazard predictions, nucleotide conservation predictions, ClinVar significance records, HGMD records, OMIM records, and pathogenicity predictions. Non-benign homozygous or heterozygous variants matching the inheritance pattern were selected as candidates. The VarSome website was then used to predict the pathogenicity of these variants, including SNVs and indels. Likely pathogenic and pathogenic variants of *IKBKG* were identified as potential IP-causing variants.

### CNVs detection based on local de novo assembly

As demonstrated in Fig. 2b, barcodes of reads aligned to non-homologous reference regions were collected. Reads sharing these barcodes were then retrieved and assembled. The CNV detection strategy based on local de novo assembly was implemented using Supernova (version 2.1.1) and SPAdes. The analysis based on Supernova was conducted with an open-source software named “stlfr2supernova_pepeline” (https://github.com/BGI-Qingdao/stlfr2supernova_pipeline) according to the user’s manual. Meanwhile, the main parameters of SPAdes were ‘--careful --only-assembler -t 16’. The assembly results were then aligned separately to the human genome (hg19) using the QUAST software (version 5.1.0rc1) with the parameters ‘-t 20 --min-identity 95 --min-contig 500’. Based on the alignment results, discontinuous or misassembled assemblers were selected as potential CNVs. Subsequently, assemblers with breakpoints caused by Ns were excluded. For the remaining assemblers, reads passing through the breakpoint were extracted, and their co-barcoded long DNA fragments were traced. The distribution patterns of all reads belonging to these long DNA fragments were then analyzed. Finally, assemblers with no other reads distributed in the deletion region were considered candidate CNVs.

### MLPA, PCR, gel electrophoresis, and Sanger sequencing

SNVs and indels were validated by PCR and Sanger sequencing, while CNVs were confirmed by MPLA using MLPA probemix P073-A1 (MRC Holland, Holland) and long-range PCR. Capillary electrophoresis was performed on ABI3500 (Life Technologies), and analyzed using Coffalyser (MRC Holland) or Gene Mapper software (Life Technologies).

### Reporting summary

Further information on research design is available in the Nature Research Reporting Summary linked to this article.

### Supplementary information

Supplementary information

Reporting summary

## Data Availability

The data that support the findings of this study have been deposited in the Genome Sequence Archive for Human with the accession code HRA006663.

## Code availability

The basic analysis pipelines of the stLFR sequencing data are publicly available on GitHub (https://github.com/MGI-tech-bioinformatics/stLFR_V1.3). The detection analysis of SNVs, indels, and CNVs that could distinguish the *IKBKG* gene from its pseudogene was based on open source software and is detailed in Methods and Supplementary Methods.
