# Supplementary material for: An efficient molecular genetic testing strategy for incontinentia pigmenti based on single-tube long fragment read sequencing
Source: NPJ Genom Med. 2024 May 29;9:32. doi: 10.1038/s41525-024-00421-z (PMC11137062; doi:10.1038/s41525-024-00421-z)
Supplement: Supplementary file 1 — Supplementary information [file 41525_2024_421_MOESM1_ESM.pdf]

Supplementary methods

The SNVs and indels in the *IKBKG* gene are detected using the following analysis procedures based on reconstructed references. For *IKBKGP1*, the reference genome and dbSNP files should be replaced.

1. Align to reconstructed references based on BWA (version 0.7.17)

   bwa mem -t 2 -M -R '@RG\tID:read_groupID \tSM:sample\tPL:COMPLETE' Ref_Reconstruction.IKBKG.chrX_153769419_153841350.fa *1.fq.gz *2.fq.gz | samtools view -S -b -o *output.mem.bam -

   samtools sort -@ 6 -m 768M -o *output.mem.sort.bam *output.mem.bam

   samtools index *output.mem.sort.bam *output.mem.sort.bai

2. Mark RCR duplication by picard (version 1.98)

   java -Xms3g -Xmx3g -Djava.io.tmpdir=javatmp -jar MarkDuplicates.jar INPUT=*.mem.sort.bam OUTPUT=*.mem.sort.Markdup.bam METRICS_FILE=*.metrics VALIDATION_STRINGENCY=SILENT

   samtools index *.mem.sort.Markdup.bam *.mem.sort.Markdup.bai

3. BQSR

   java -Xms3g -Xmx3g -Djava.io.tmpdir=javatmp -jar gatk-package-4.1.4.0-local.jar BaseRecalibrator -R Ref_Reconstruction.IKBKG.chrX_153769419_153841350.fa -I *.mem.sort.Markdup.bam -O *.mem.sort.Markdup.recal.table --known-sites dbSNP_b151_GRCh37p13.chrX_153769419_153841350.vcf

   java -Xms3g -Xmx3g -Djava.io.tmpdir=javatmp -jar gatk-package-4.1.4.0-local.jar ApplyBQSR -R Ref_Reconstruction.IKBKG.chrX_153769419_153841350.fa -I *.mem.sort.Markdup.bam -O *.mem.sort.Markdup.bqsr.bam -bqsr *.mem.sort.Markdup.recal.table --static-quantized-quals 10 --static-quantized-quals 20 --static-quantized-quals 30 --static-quantized-quals 40 --emit-original-quals

4. Variants calling and filtration based on GATK

   java -Xms3g -Xmx3g -Djava.io.tmpdir=javatmp -jar gatk-package-4.1.4.0-local.jar HaplotypeCaller -I *.mem.sort.Markdup.bqsr.bam -O *raw.vcf -R Ref_Reconstruction.IKBKG.chrX_153769419_153841350.fa --dbsnp dbSNP_b151_GRCh37p13.chrX_153769419_153841350.vcf >> *log

java -Xms3g -Xmx3g -Djava.io.tmpdir=javatmp -jar gatk-package-4.1.4.0-local.jar SelectVariants -R Ref_Reconstruction.IKBKG.chrX_153769419_153841350.fa -V *raw.vcf --select-type-to-include SNP -O *raw.SNP.vcf

java -Xms3g -Xmx3g -Djava.io.tmpdir=javatmp -jar gatk-package-4.1.4.0-local.jar SelectVariants -R Ref_Reconstruction.IKBKG.chrX_153769419_153841350.fa -V *raw.vcf --select-type-to-include INDEL -O *raw.INDEL.vcf

java -Xms3g -Xmx3g -Djava.io.tmpdir=javatmp -jar gatk-package-4.1.4.0-local.jar VariantFiltration -V *raw.SNP.vcf -O *raw.SNP.filter.vcf -R Ref_Reconstruction.IKBKG.chrX_153769419_153841350.fa --filter-expression "QD<2.0 || MQ<40.0 || FS>60.0 || MQRankSum<-12.5 || ReadPosRankSum<-8.0" --filter-name "StandardFilter"

java -Xms3g -Xmx3g -Djava.io.tmpdir=javatmp -jar gatk-package-4.1.4.0-local.jar VariantFiltration -V *raw.INDEL.vcf -O *raw.INDEL.filter.vcf -R Ref_Reconstruction.IKBKG.chrX_153769419_153841350.fa --filter-expression "QD<2.0 || FS>200.0 || SOR > 10.0 || MQRankSum<-12.5 || ReadPosRankSum<-8.0" --filter-name "StandardFilter"

java -Xms1g -Xmx1g -Djava.io.tmpdir=javatmp -jar gatk-package-4.1.4.0-local.jar MergeVcfs -I *raw.SNP.filter.vcf -I *raw.INDEL.filter.vcf -O *final.vcf

Supplementary Table 1. Comparison of different molecular testing techniques for IP

Technology	long range PCR	MLPA	stLFR
Targeted or whole genome	Targeted	Targeted	Whole genome
Detected variant type	SNVs and CNVs	CNVs	SNVs, InDels, CNVs and other SVs
Cost	Low	Medium	High
Validation required	No	Yes	Yes
Pseudogene differentiation	Yes	No	Yes
Analysis methods	Sanger sequencing	Fragment analysis	Sequencing

Supplementary figures

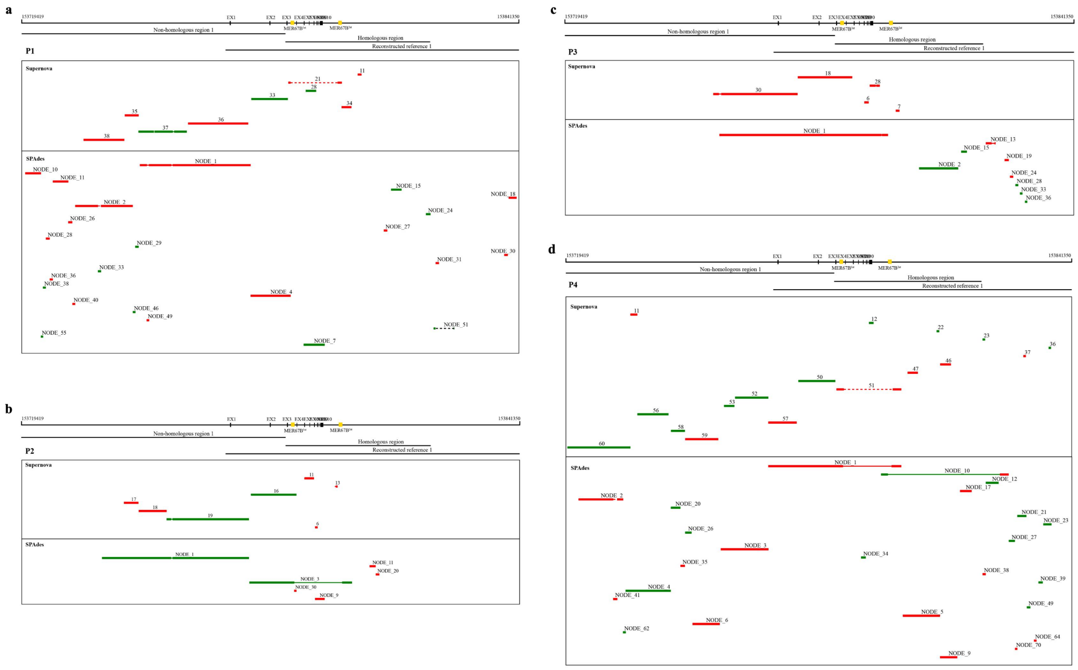

Supplementary Figure 1. Assemble results based on Supernova and SPAdes.

Each red or green line represents an assembler with a specific index indicated above. Red lines indicate assemblers that match the reference genome, while green lines indicate assemblers that match the reverse complementary sequence of the reference genome. Dashed lines represent assembly sequences (scaffolds) that cannot be mapped to the reference genome. Solid lines represent assembly sequences (contigs) that may have a structural deletion compared to the reference genome.

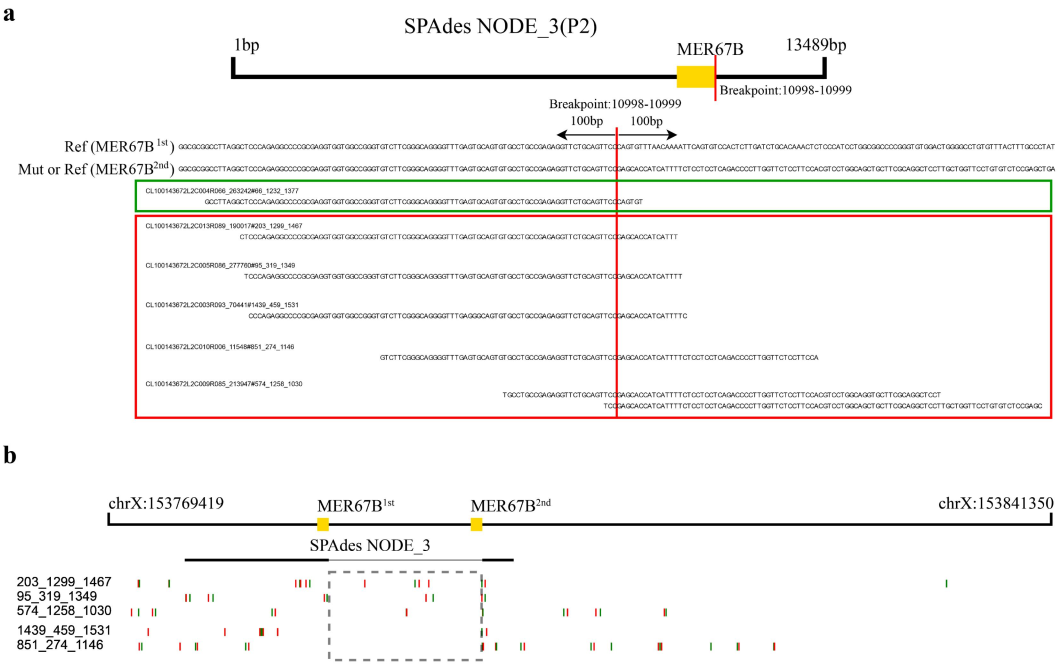

Supplementary Figure 2. Distribution of supporting long fragment reads passing through the breakpoints in P2.

(a) Six reads passing through the breakpoint. Of these, one aligns to the MER67B1st sequence (Ref MER67B1st) and five align to either the *IKBKG*$^{\triangle 4\text{-}10}$ mutant sequence (Mut) or the MER67B^{2nd} sequence (Ref MER67B2nd). (b) Distribution of co-barcoded reads. Each red or green short vertical line represents a read. Red ones represent the first segments in the template, and green ones represent the last segments in the template. The deletion region is indicated by a dashed box.

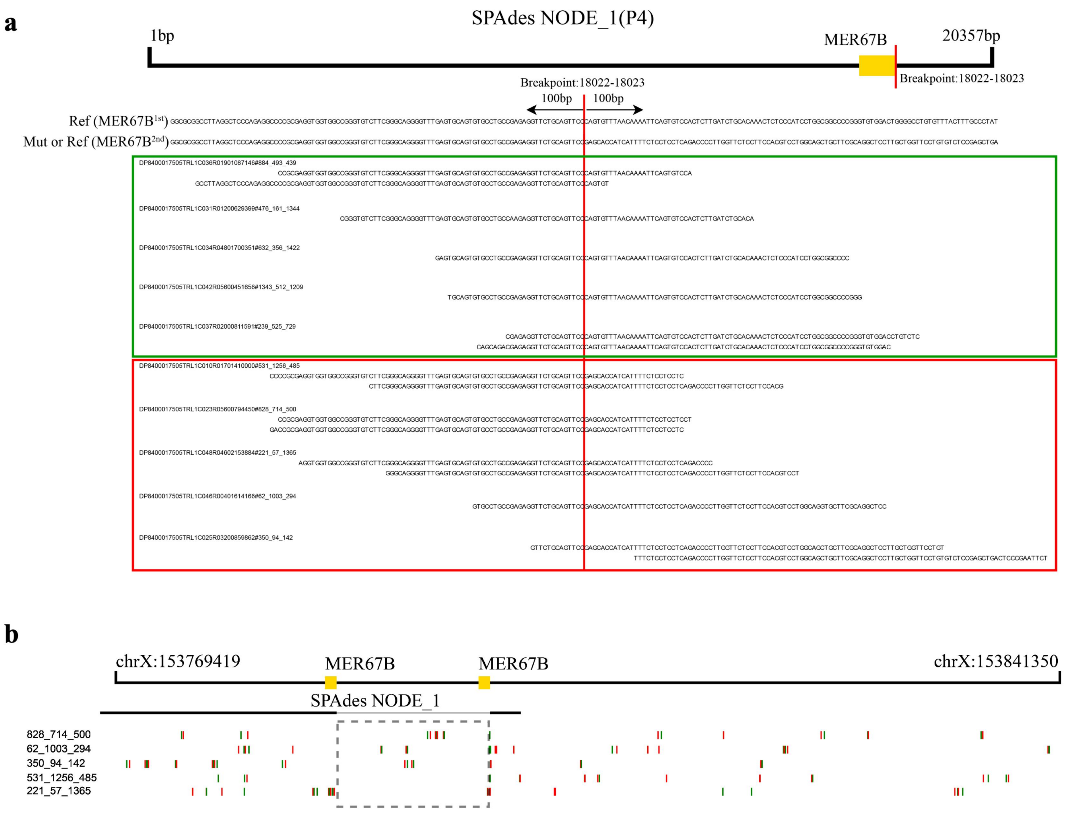

Supplementary Figure 3. Distribution of supporting long fragment reads passing through the breakpoint in P4.

(a) Ten reads passing through the breakpoint, with five aligning to Ref MER67B1st and five aligning to either Mut or Ref MER67B2nd. (b) Distribution of co-barcoded reads. Each red or green short vertical line represents one read. The deletion region is indicated by a dashed box.
